# The Circadian Rhythm Gene *Arntl2* Is a Metastasis Susceptibility Gene for Estrogen Receptor-Negative Breast Cancer

**DOI:** 10.1371/journal.pgen.1006267

**Published:** 2016-09-22

**Authors:** Ngoc-Han Ha, Jirong Long, Qiuyin Cai, Xiao Ou Shu, Kent W. Hunter

**Affiliations:** 1 Laboratory of Cancer Biology and Genetics, National Cancer Institute, National Institutes of Health, Bethesda, Maryland, United States of America; 2 Division of Epidemiology, Department of Medicine, Vanderbilt Epidemiology Center, Vanderbilt-Ingram Cancer Center, Vanderbilt University School of Medicine, Nashville, Tennessee, United States of America; Stanford University School of Medicine, UNITED STATES

## Abstract

Breast cancer mortality is primarily due to metastasis rather than primary tumors, yet relatively little is understood regarding the etiology of metastatic breast cancer. Previously, using a mouse genetics approach, we demonstrated that inherited germline polymorphisms contribute to metastatic disease, and that these single nucleotide polymorphisms (SNPs) could be used to predict outcome in breast cancer patients. In this study, a backcross between a highly metastatic (FVB/NJ) and low metastatic (MOLF/EiJ) mouse strain identified *Arntl2*, a gene encoding a circadian rhythm transcription factor, as a metastasis susceptibility gene associated with progression, specifically in estrogen receptor-negative breast cancer patients. Integrated whole genome sequence analysis with DNase hypersensitivity sites reveals SNPs in the predicted promoter of *Arntl2*. Using CRISPR/Cas9-mediated substitution of the MOLF promoter, we demonstrate that the SNPs regulate *Arntl2* transcription and affect metastatic burden. Finally, analysis of SNPs associated with *ARNTL2* expression in human breast cancer patients revealed reproducible associations of *ARNTL2* expression quantitative trait loci (eQTL) SNPs with disease-free survival, consistent with the mouse studies.

## Author Summary

Estrogen receptor-negative (ER-) breast cancer is a highly malignant form of breast cancer with poor prognosis. Like most solid tumors, the mortality associated with ER- breast cancer is due to the development of secondary tumors, or metastases, in vital organs distant from the original breast tumor. ER- breast tumors, particularly those also lacking HER (human epidermal growth factor receptor) expression, are particularly deadly because unlike ER+ or HER+ breast cancers, targeted therapies have not yet been developed that can effectively reduce or eliminate tumor cells that have disseminated throughout the patient. Therefore, better understanding of the etiology of metastasis in ER- patients would potentially have a large clinical benefit by providing new targets to eradicate single tumor cells before they develop into metastases. A better understanding of metastasis etiology may also provide methods to prevent the conversion of these disseminated cells into life-threatening lesions. In this study, we demonstrate that a commonly used model of metastatic breast cancer is capable of identifying genes that play a role in the metastatic progression of ER- breast cancers. Furthermore, we identify the circadian rhythm gene, *Arntl2*, as a gene associated with inherited susceptibility for the development of metastatic lesions. These studies provide additional information regarding the mechanisms associated with metastasis in ER- breast cancers.

## Introduction

Breast cancer is the most common form of malignancy in women and is the second leading cause of cancer-related death for women in the United States [1]. Mortality for breast cancer, like most solid cancers, is due not to the primary tumor but instead to metastases, the secondary tumors that arise in distant anatomical sites from cells that have disseminated from the original tumor mass. If the tumor remains localized, the five-year survival rate for breast cancer approaches 99%, suggesting that current clinical interventions for localized breast cancer are highly effective. In contrast, for women with distant metastatic disease the survival rate plummets to 26% [1], emphasizing the need for new approaches to deal with metastatic lesions.

Metastatic breast cancer therefore remains a significant public health problem. It has been estimated that approximately 12% of women in the United States will be diagnosed with breast cancer during their lifetime [1]. At present, almost 240,000 women are diagnosed with breast cancer annually, and approximately 3 million women are living with breast cancer in the United States [1]. Although only six percent (N~ 14,000) of new cases have metastatic involvement at the presentation of breast cancer, approximately 30% of women without evidence of disseminated disease may develop metastatic lesions later in life [2]. As a result, every year in the United States approximately 40,000 women die due to their metastatic breast cancer. While earlier detection and application of anti-metastatic adjuvant therapies have contributed to the increased survival of women over the past decades, metastatic disease remains a significant problem due to tumor cell dormancy and resistance of established metastatic lesions to therapy. Major improvements in patients’ long term survival will therefore come in part from better prevention of metastatic disease through enhanced adjuvant therapy and improved targeting of established metastasis.

Advances in a variety of genomic tools have greatly enhanced our understanding of primary breast cancer. Next generation sequencing studies have provided detailed understanding of the common mutational events that drive tumorigenesis in breast cancer [3]. In addition, gene expression studies of primary tumors have revealed that the histochemically defined ER^+^ or ER^-^ classes can be further subdivided based on molecular profiles and that these additional classes have distinct prognostic outcomes [4, 5]. Targeted therapies exist for the luminal and HER2+ (human epidermal growth factor receptor 2) subtypes of human breast cancers which significantly improve patient outcome by elimination of occult tumor cells through adjuvant therapies, thereby reducing the incidence of metastatic disease. Analysis of genome-wide primary tumor gene expression has also been successfully used to predict prognosis of breast cancer (ex. [4]). This approach may allow for better application of targeted therapeutics to at-risk patients which could improve outcome while reducing unnecessary treatment-associated morbidity for patients with low risk of metastatic disease.

Despite these successes, metastatic disease continues to be a major hurdle, particularly for patients with ER^-^ tumors which include both the basal and triple negative (estrogen- and progesterone receptor-negative, HER2-negative) subtypes. These tumors have the worst prognosis among all of the breast cancer subtypes with rapid relapse after diagnosis and poor overall survival [6]. At present, no targeted therapeutic agents are available to combat progression of ER^-^ tumors. As a result, most patients with ER^-^ tumors are routinely treated with conventional chemotherapeutics, despite their considerable side effects, in an effort to improve outcome. ER^-^ breast cancer patients may therefore benefit most from in-depth investigations into the etiology of metastatic disease through identification of better targets to reduce disseminated cells and subclinical lesions prior to the development of pathologic metastases.

The FVB/NJ-TgN(MMTV-PyMT)^634Mul^ (hereafter, MMTV-PyMT) genetically engineered mouse is a highly aggressive, metastatic model of mammary tumors [7]. Expression analysis of this model suggests that it most closely resembles the Luminal subtype of human breast cancer. In earlier studies [8, 9], our laboratory has used this model to identify polymorphic genes within the mouse genome that influence metastatic progression [10, 11]. Studies of several of these genes have indicated that the human orthologs of the mouse metastasis susceptibility genes are significantly associated with progression only in ER^+^ patients, consistent with the assignment of the MMTV-PyMT as a luminal cancer model [12]. However, our laboratory has recently demonstrated that the genetic background of this animal not only influences metastatic susceptibility but also the gene expression patterns used to assign molecular subtype [13]. In this study, we further extend those results to demonstrate that crosses between the MMTV-PyMT model and the Asian-derived mouse strain MOLF/EiJ generate gene signatures that are prognostic in ER^-^ rather than ER^+^ breast cancers. Moreover, these analyses led to the identification of the circadian rhythm gene, *Arntl2*, as a metastasis susceptibility gene, suggesting that circadian rhythms play an important role not only in the etiology, but also in progression of the most aggressive form of breast cancer.

## Results

### Quantitative trait loci for tumor latency, growth and metastatic progression maps to distal chromosome 6

**Fig 1:**
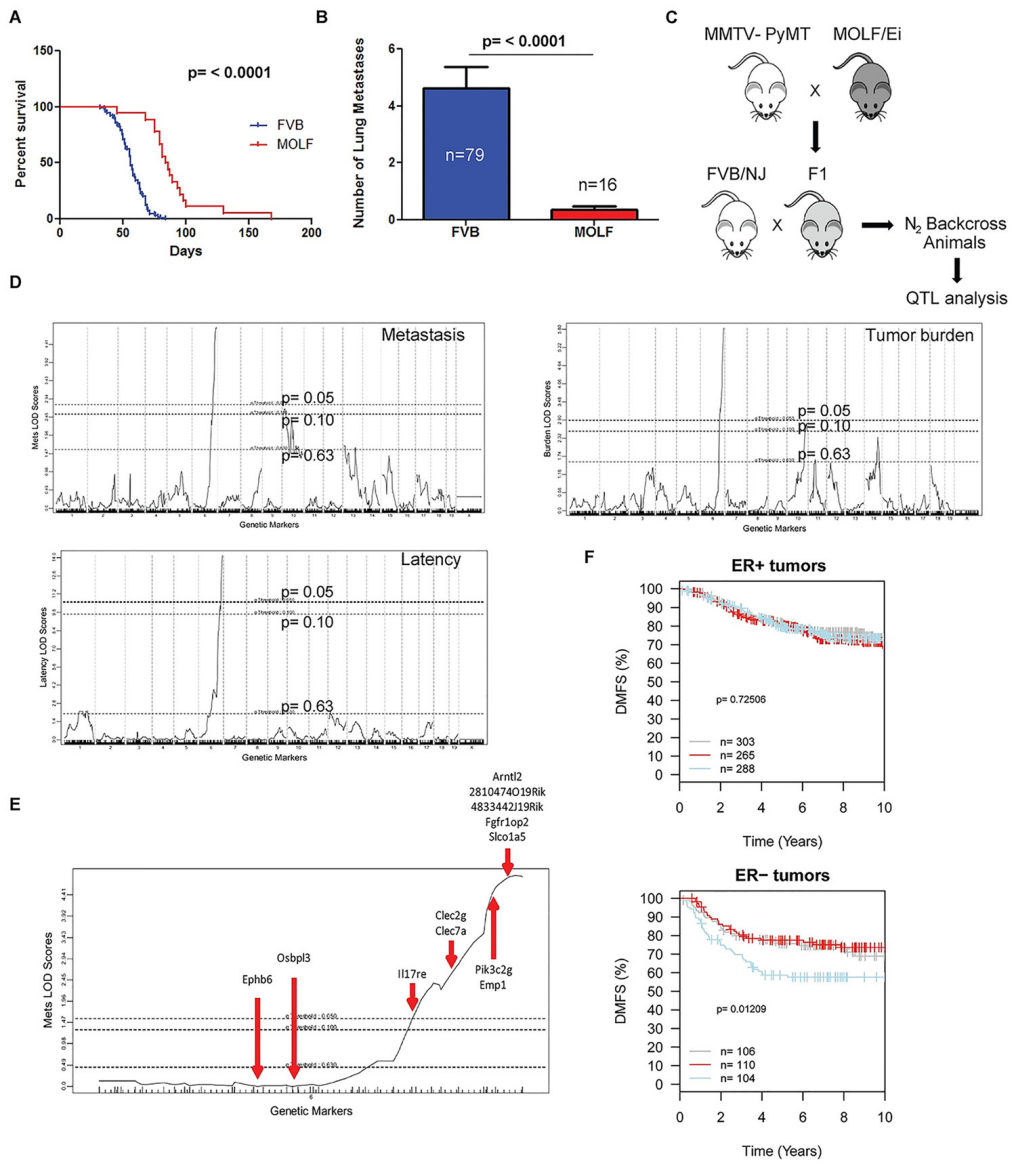
Distal chromosome 6 is a metastasis modifier. (A) Survival plot of FVB/NJ x (MOLF/EiJ x MMTV-PyMT) mice. p-value calculated by Log-Rank test. FVB = FVB/NJ x MMTV-PyMT; MOLF = MOLF/EiJ x MMTV-PyMT. (B) Surface metastasis count of FVB/NJ x (MOLF/EiJ x MMTV-PyMT) mice. p-value calculated by Mann-Whitney test. FVB = FVB/NJ x MMTV-PyMT; MOLF = MOLF/EiJ x MMTV-PyMT. (C) Depiction of breeding scheme. (D) LOD score plot for metastasis susceptibility, latency and tumor burden loci in the backcross. The y-axis represents the LOD score observed across the genome. The mouse chromosomes are presented head-to-tail on the x-axis. The lower horizontal dashed line represents the suggestive LOD score for a quantitative trait locus after correction for multiple testing by permutation testing. The upper dashed line is the significance threshold after permutation testing. (E) Approximate location of metastasis-associated eQTL candidate genes on distal chromosome 6. (F) The Gene expression-based Outcome for Breast Cancer Online (GOBO) database was queried for the eQTL genes on distal chromosome 6. Distant metastasis-free survival (DMFS) plotted as Kaplan-Meier curves for patients with ER+ and ER- tumors expressing high (blue), intermediate (red), or low (gray) levels of the eQTL genes.

Outcrosses were previously performed between the highly metastatic MMTV-PyMT model of breast cancer and members of different branches of the mouse phylogenetic tree to identify inbred strains harboring genetic tumor modifiers [14]. The strain MOLF/EiJ was found to have one of the most significant effects on tumorigenesis, extending tumor latency (Fig 1A) as well as suppressing tumor growth and development of pulmonary metastatic lesions (Fig 1B). To map the chromosomal regions associated with these phenotypes, an [FVB/NJ x (MOLF/EiJ x MMTV-PyMT)]N_2_ backcross (N = 171) was generated and genotyped using the Illumina Mouse Medium Density Linkage Panel (Fig 1C). A significant association was observed for all three phenotypes with distal chromosome 6 after genome-wide permutation correction (Fig 1D) [15]. Additional suggestive peaks for tumor growth and metastasis were observed on distal and proximal chromosome 10, respectively. In contrast to previous genetics studies performed with the MMTV-PyMT model, the significant distal chromosome 6 peaks for all three tumor phenotypes were superimposable and peaked at the very distal end of the chromosome. This suggests the possibility of a common modifier for all three phenotypes in the MOLF/EiJ strain, in contrast to earlier studies where the modifiers of the individual tumor phenotypes were present on separate chromosomes.

### QTL candidate genes predict patient survival in ER-negative breast cancer

Based on the observation that the majority of single nucleotide polymorphisms associated with disease in humans are non-coding [16], we performed an eQTL analysis of mammary tumors from the [FVB/NJ x (MOLF/EiJ x MMTV-PyMT)]N_2_ backcross in order to identify potential candidate genes. Tumors from 134 animals were arrayed on Affymetrix ST 1.0 arrays (GSE48566; [13]) and screened for genes in the chromosome 6 interval that were associated with distant metastasis-free survival. Twelve genes on chromosome 6 were found to be significantly associated (p <0.001 and FDR <0.05) with the presence or absence of metastasis in the MOLF/EiJ backcross (Fig 1E and S1 Table). To determine whether these genes play a role in human breast cancer progression and metastasis, the human orthologs were identified and used as a gene signature to stratify breast cancer patients using GOBO. The individual genes within the signature were weighted using the mouse-derived hazard ratios to require similar direction and relative strength in the human patient cohort. As can be observed in Fig 1F, the weighted gene signature was able to discriminate outcome in human patients, consistent with one or more of the genes being associated with progression in both species. Surprisingly, despite the fact that the MMTV-PyMT tumor system is thought to be a model of ER+ tumors [8, 9], stratification of the patients by hormone receptor status demonstrated that the ability to discriminate metastasis-free survival was specific for ER- patients. This result is consistent with earlier observations that genetic background can significantly influence subtype assignments that are based on gene expression methods [13].

### ARNTL2 levels significantly influence mammary tumor metastasis

**Fig 2:**
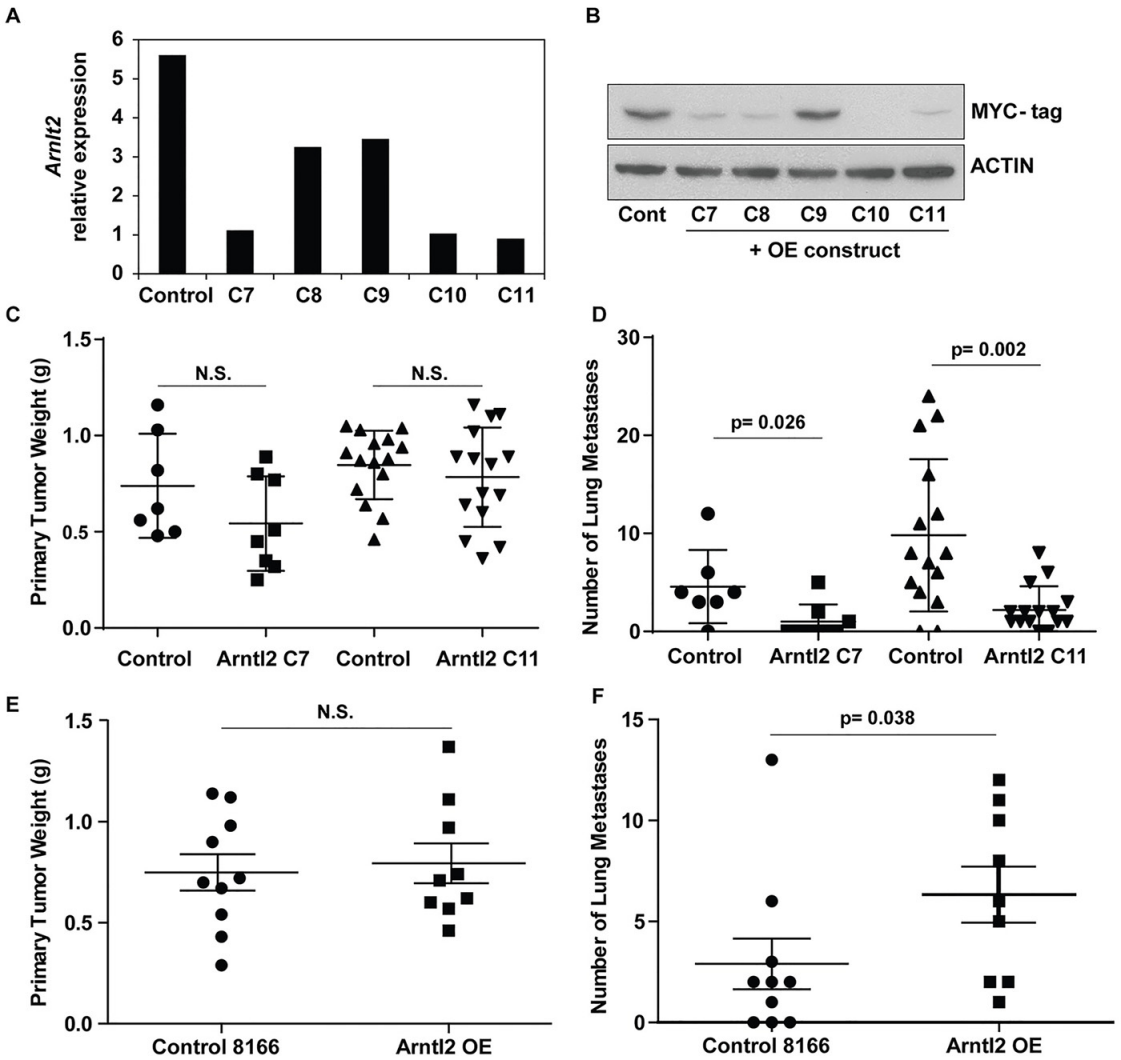
Effect of Arntl2 expression *in vivo*. (A) Relative *Arntl2* mRNA expression in 4T1 cells with control (scramble) and *Arntl2* shRNA cell lines. (B) Protein expression of ARNTL2 in transiently transfected HEK293 cells with shRNA and overexpression vector expressing myc-tag. Actin is the loading control. (C) Weight of primary tumor from orthotopically injected BALB/cJ mice with 4T1 control and C7 and C11 *Arntl2* shRNA constructs. N.S. = not significant. (D) Count of surface pulmonary nodules from (C). P-value calculated by two-tailed Mann-Whitney test. (E) Primary tumor weight of *Arntl2* overexpressing 4T1 cells. N.S. = not significant. (F) Lung metastases from (E), p-value calculated by one-tailed Mann-Whitney test.

To begin to dissect the contributions of individual genes within the QTL interval, *Arntl2* was selected due to its position at the apex of the QTL peak and it having the most significant association with metastasis (based on the p-value) among the candidate genes (Fig 1E). To assess whether *Arntl2* expression affects phenotypes related to tumor burden as well as metastasis *in vivo*, *Arntl2* expression was knocked down in the 4T1 mouse mammary cell line using five shRNA constructs against murine *Arntl2* (Fig 2A). Since we were unable to validate a reliable antibody against mouse ARNTL2, we transiently transfected HEK293 cells with each of the five shRNA constructs along with a myc-expressing *Arntl2* overexpression plasmid to verify the shRNA efficacy. Similar to the mRNA levels in 4T1 cells, significant reduction in protein ARNTL2 levels were achieved by observation of myc expression (Fig 2B). To test the effect of *Arntl2* knockdown on cell phenotypes, *in vitro* cell proliferation and migration assays were performed. No significant differences were observed in either *in vitro* assay, indicating that *Arntl2* does not alter these *in vitro* phenotypes (S1A and S1B Fig). To specifically assess metastatic potential *in vivo*, control- and *Arntl2* shRNA-transduced 4T1 cells were injected orthotopically into the fourth mammary fat pad of syngeneic BALB/cJ mice. The mice were assessed 28 days post-injection for primary tumor weight and pulmonary metastasis burden. Knockdown of *Arntl2* did not produce a significant change in primary tumor growth (Fig 2C). However, reduction of *Arntl2* expression significantly decreased the number of pulmonary metastatic nodules (Fig 2D). Similarly, overexpression of ARNTL2 in 4T1 mouse mammary tumor cells (S1C and S1D Fig) increased pulmonary metastases without significantly affecting primary tumor growth (Fig 2E and 2F). Therefore, these *in vivo* data establish ARNTL2 as a metastasis-specific modifier. Unexpectedly, the metastasis promoting effect of *Arntl2* in the 4T1 cells was the opposite of that predicted from the gene expression analysis from tumor tissue (metastasis protective). We therefore performed a number of studies to determine whether the difference between the spontaneous/endogenous tumor and cell line-based tumor studies were due to the contribution of other factors/genes within the chromosome 6 locus or due to cell line-specific artifacts in the orthotopic transplant assays.

### *Arntl2* knockout suppresses MMTV-PyMT metastatic progression in a tumor autonomous manner

**Fig 3:**
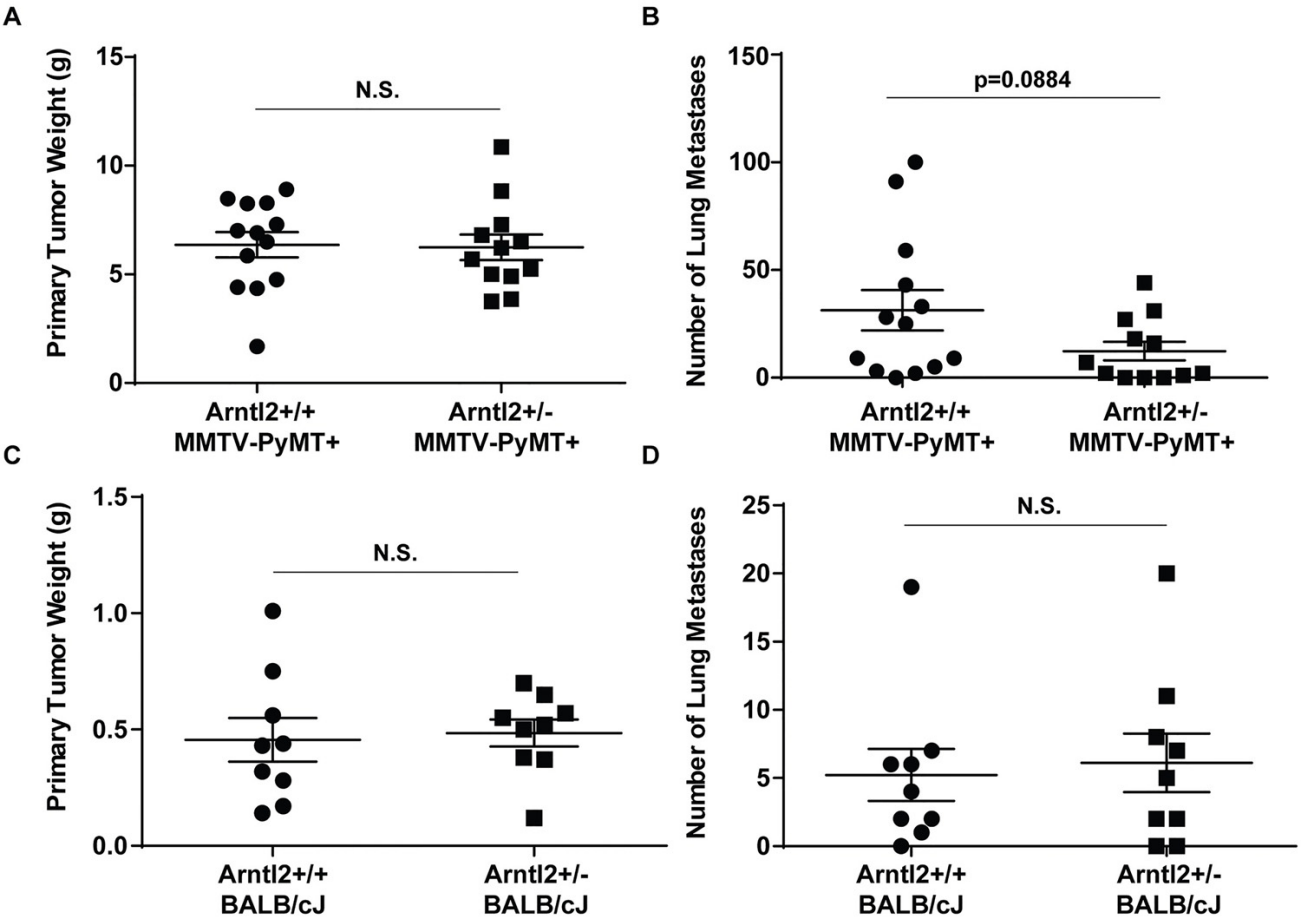
Tumor-autonomous effect of Arntl2. (A) *Arntl2*^+/+^ and *Arntl2*^-/-^ mice were bred to MMTV-PyMT+ mice to generate spontaneous primary tumors and lung metastases. At the time of euthanasia, autochthonous PyMT-induced tumors were weighed. N.S. = not significant. (B) Surface lung metastasis count of spontaneous metastases from (A). P-value calculated by two-tailed Mann-Whitney test (C) Primary tumor weight of orthotopically injected 4T1 cells into F1 hybrids from *Arntl2*^+/+^ and Arntl2^+/-^ mice crossed with BALB/cJ mice. N.S. = not significant. (D) Surface lung metastasis count of the injected mice in (C). N.S. = not significant.

The cell line-based shRNA and overexpression studies are consistent with *Arntl2* functioning as a metastasis promoting factor. However, to rule out the possibility that the prognostic phase inversion seen in the MOLF/EiJ x MMTV-PyMT cross and human patients is not due to artifact introduced by selection for *in vitro* growth, *in vivo* validation was performed. *Arntl2* knockout (KO) mice were obtained from the KOMP (Knock Out Mouse Project) repository [17] and were bred to MMTV-PyMT mice to generate *Arntl2*^+/-^; MMTV-PyMT^+^ or *Arntl2*^+/+^; MMTV-PyMT^+^ females. Animals were permitted to age for tumor initiation and progression, and pulmonary lung metastases were enumerated after euthanasia. In agreement with our previous data, suppression of pulmonary metastases was observed in the *Arntl2*^+/-^; MMTV-PyMT^+^ compared to the *Arntl2*^+/+^; MMTV-PyMT^+^ animals (Fig 3B) without affecting the primary tumor (Fig 3A). Taken together, the cell line and *in vivo* animal studies suggest that *Arntl2* is a metastasis-promoting inherited susceptibility factor. Furthermore, these studies indicate that the difference between the prognostic effect of *Arntl2* alone and that of the entire chromosome 6 locus is due to additional factors acting either additively or epistatically with *Arntl2*.

Previous efforts in our laboratory have demonstrated that metastasis susceptibility factors can function as tumor autonomous factors [18–21] or by a tumor autonomous mechanism that results in engagement of stromal cells [10]. To test whether *Arntl2*-mediated metastasis promotion indirectly involves stromal or immune components other than the tumor cells, the *Arntl2*^-/-^ mice were bred to BALB/cJ animals to make F1 hybrids. This allows for orthotopic injection of BALB/cJ-derived cell lines (4T1) without an immune rejection. Unmanipulated 4T1 mammary tumor cells were then implanted into the *Arntl2*^+/+^ x BALB/cJ or *Arntl2*^+/-^ x BALB/cJ mice and pulmonary metastases enumerated after tumor growth and progression. No difference was observed between the wildtype or heterozygous knockout mice (Fig 3C and 3D) suggesting a tumor-autonomous effect on metastasis progression. While the effect of reduction of ARNTL2 in stromal cells or cells other than the tumor cells cannot be completely disregarded, these results indicate that the major role of *Arntl2* in metastasis is due to its effect in the tumor cells themselves.

### MOLF/EiJ Arntl2 promoter polymorphisms reduce Arntl2 expression and suppress metastases

To identify polymorphisms that might be responsible for the difference in *Arntl2*-mediated metastasis susceptibility, whole genome sequencing from the MOLF/EiJ genome (Doran et al, Genome Biology, in press) was performed. No sequence variants that distinguish FVB/NJ and MOLF/EiJ were identified in the *Arntl2* coding region. The genomes were then examined to identify SNPs in regulatory regions that might alter *Arntl2* expression. DNase hypersensitivity sites (DHS) from the 3134 mammary tumor cell line [22] were screened to identify potential gene regulatory regions near *Arntl2*. Two hypersensitivity sites were identified approximately 10 and 12 kb upstream of the predicted transcriptional start site for the *Arntl2* Refseq transcripts (S2A Fig). Interestingly, no DHS was observed at the Refseq transcriptional start site, suggesting that the two upstream hypersensitivity sites were associated with the *Arntl2* promoter rather than an enhancer element. Consistent with this possibility, searches of the Ensembl database identified an EST (expressed sequence-tag) that spans the proximal DHS and the first coding exon (S2A Fig). RT-PCR in 4T1 cells confirmed the predicted spliced product, further suggesting that the two DHS are part of the *Arntl2* proximal promoter region (S2B Fig). Overlapping these data, ten SNPs were identified within the putative proximal promoter DHS that distinguished FVB/NJ from MOLF/EiJ (S2A Fig). The SNPs were confirmed by direct target sequencing of this region in FVB/NJ and MOLF/EiJ DNA. In addition to the SNPs in the putative proximal promoter DHS, 12 polymorphisms in the 3’UTR of *Arntl2* were also identified. While these SNPs could affect miRNA binding and therefore transcript expression, we focused on those that could alter transcription factor and/or chromatin regulator binding.

**Fig 4:**
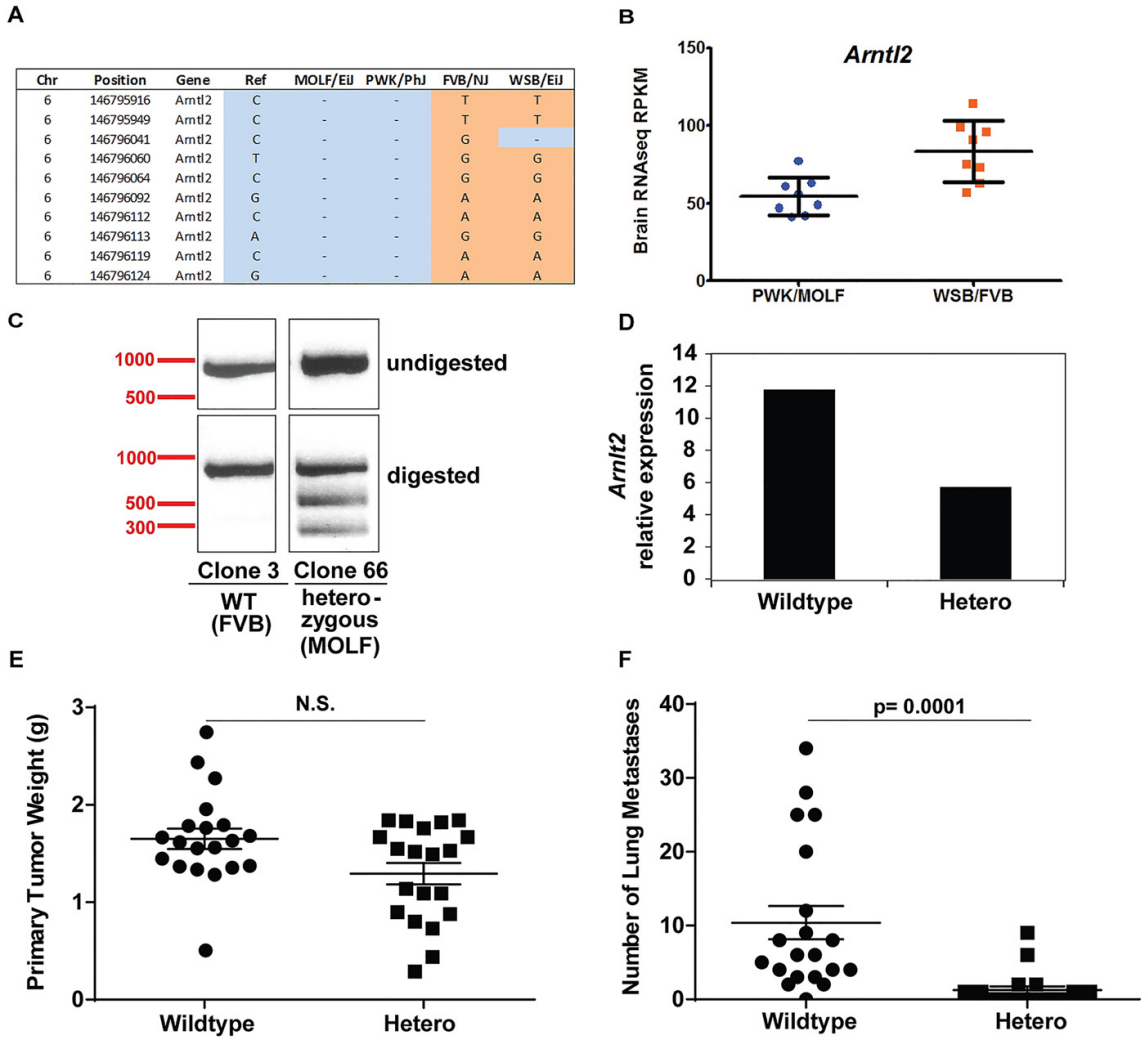
Genome editing of the *Arntl2* promoter. (A) Haplotypes for the *Arntl2* putative promoter from the FVB, MOLF, PWK and WSB inbred strains. Brain RNAseq data shows that wild-derived PWK/PhJ carries the same haplotype as MOLF/EiJ, and WSB/EiJ shares 9 of the 10 FVB/NJ SNPs at this location. (B) Relative expression of *Arntl2* in brain RNA from the PWK and WSB strains. (C) Confirmation of genomically-edited 6DT1 cells. (D) Relative mRNA expression of *Arntl2* in CRISPR-engineered 6DT1 cells as measured by qPCR. (E) Primary tumor weight of injected CRISPR-engineered WT and heterozygous substituted cells in FVB mice. N.S. = not significant. (F) Surface lung metastasis count on injected cells in (E). P-value calculated by two-tailed Mann-Whitney test.

To assess the potential effect of these SNPs on *Arntl2* expression, publicly available RNA-seq and SNP data was examined. Brain RNAseq data (http://csbio.unc.edu/gecco/) shows that wild-derived PWK/PhJ carries the same haplotype as MOLF/EiJ, and WSB/EiJ shares 9 of the 10 FVB/NJ SNPs at this location (Fig 4A). The data demonstrated ~30% lower *Arntl2* expression by PWK compared to WSB, supporting the hypothesis that these SNPs affect *Arntl2* expression (Fig 4B). To assess these SNPs *in vivo* in our cell lines, CRISPR/Cas9- mediated replacement of the ten SNPs across the putative promoter DHS was performed. The promoter replacement was performed in 6DT1, a cell line derived from the MMTV-myc FVB-based genetically engineered model, to be certain that any effects observed were not due to a 4T1-specific cell line artifact. Single cell clones were assessed for successful integration of the MOLF region by PCR amplification and subsequent MOLF SNP-specific restriction enzyme digestion (Fig 4C), followed by Sanger sequencing of the region. As predicted by the wild-derived mouse brain RNAseq data (Fig 4B), replacement of the FVB/NJ DHS allele with the MOLF/EiJ allele resulted in a decreased expression of *Arntl2* mRNA (Fig 4D). Orthotopic implantation of the CRISPR-substituted cell line also resulted in a suppression of pulmonary metastasis without significant alteration of primary tumor growth, consistent with the shRNA experiments in the 4T1 cell line (Fig 4E and 4F). Taken together, these data indicate that the differential expression of *Arntl2* observed between FVB/NJ and MOLF/EiJ is most likely due to polymorphisms in the promoter of *Arntl2* that change its expression and modulate metastasis burden.

### Polymorphisms associated with expression of human *ARNTL2* are correlated with prognosis of ER- breast cancer in human

Validation of *ARNTL2* as a *bone fide* metastasis susceptibility gene was performed by association studies in two human ER^-^ breast cancer patient cohorts. Since the effect in mouse was seen at the gene expression level, we performed a human expression quantitative trait locus (eQTL) analysis to see whether inherited variation in human *ARNTL2* expression was associated with prognosis in accordance with our hypothesis. *ARNTL2* eQTL SNPs were selected based on linear regression analyses of all SNPs within breast tissue exhibiting *ARNTL2* expression using two large public data sets: GTEx (Genotype-Tissue Expression, gene expression data from 183 normal breast tissues) and TCGA (The Cancer Genome Atlas, gene expression data from 168 ER negative breast cancer tissues). Significant eQTL SNPs selected from the GTEx (P<0.05) or from TCGA (P<1e-7, a more stringent criterion, was applied due to the concern that tumor characteristics may influence gene expression) were evaluated for their association with disease free-survival (DFS) using a Cox regression model. For SNPs in strong LD (linkage disequilibrium) with each other (r^2^>0.8), only one SNP was selected for genotyping using the Sequenom platform in the validation cohort. Covariates adjusted in the analysis were age at diagnosis, PR status, TNM stage, and cancer treatment. Fourteen SNPs were examined for DFS in a cohort of 726 ER^-^ patients included in a genome-wide association study (GWAS) and in an independent set of 1032 ER^-^ patients with targeted Sequenom-based genotyping. The minor allele of SNP rs4964008 showed a consistent association with DSF in both cohorts, and the association reached significance when the two studies were combined (Table 1). Based on the data from GTEx, alternative allele C (coded on human reference hg19 forward strand) of SNP rs4964008 was associated with a lower expression level of *ARNTL2* with a p-value of 0.0098. This allele was also associated with better survival in the present study, with a HR (95% CI) of 0.71 (0.52–0.97) and p-value of 0.03. None of the other SNPs examined were significantly associated with DFS. While not conclusive, these results are consistent with the hypothesis that inherited variants that influence *ARNTL2* expression are also associated with prognosis in humans, as they are in mice.

**Table 1:** Human eQTL SNPs associated with ARNTL2 expression that are prognostic for ER- breast cancer.

Cohort	GWAS			Sequenom			Combined		
SNP	Hazard Ratio	95% CI	p	Hazard Ratio	95% CI	p	Hazard Ratio	95% CI	p
**rs4964008**	0.76	(0.47–1.24)	0.27	0.64	(0.42–0.96)	0.03	0.71	(0.52–0.97)	0.03

### *Arntl2* promoter SNPs alter interactions with chromatin binding proteins

The *in vivo* and allograft data demonstrated that *Arntl2* is a metastasis modifier in both mouse and human and that polymorphisms in the promoter of *Arntl2* contribute to the differential expression observed between FVB/NJ and MOLF/EiJ. We therefore examined whether the promoter polymorphisms resulted in altered binding of transcription factors or chromatin regulators using an *in vitro* pull-down assay with biotinylated FVB and MOLF promoter probes. Subsequent mass-spectrometry of the proteins associated with the promoter probes identified several chromatin modifier proteins (S1 File). Interestingly, one of the proteins was PARP1 (poly(ADP-ribose) polymerase-1), a chromatin modifier previously shown to enhance transcription via binding to gene promoters [23]. Furthermore, it was recently demonstrated that PARP1 and CTCF are involved in regulating oscillation of circadian genes between active and repressed states [24]. Binding of both proteins simultaneously to actively transcribed chromatin loci results in circadian genes moving closer to the nuclear lamina, resulting in transcriptional inhibition. In addition, bioinformatics analysis of the DHS site/promoter identified a binding site for CTCF (CCGCGNGGNGGCAG), suggesting that the SNPs could disrupt CTCF binding. These data suggested that differential binding of PARP1 and CTCF might contribute to the observed expression differences and subsequent effects of *Arntl2* on metastatic disease.

**Fig 5:**
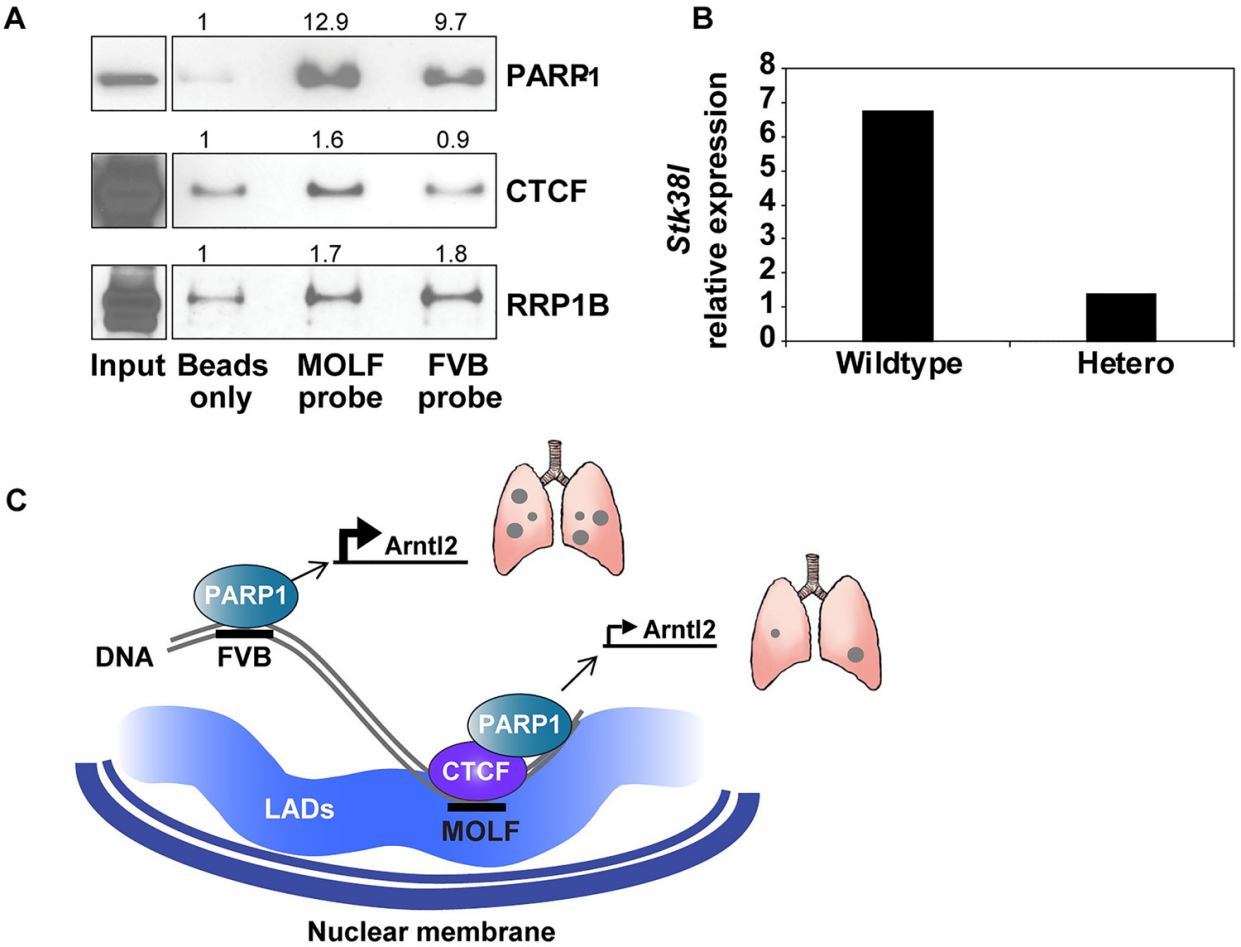
SNPs within the *Arntl2* promoter modulate chromatin binding. (A) Differential binding of MOLF and FVB promoter DNA probes to chromatin proteins. Relative densitometry compared to beads only is reported above each sample. Densitometry was calculated using ImageJ. (B) Relative mRNA expression of *Stkl3* in wildtype and heterozygous CRISPR 6DT1 cells. (C) Working model depicting differential chromatin binding of MOLF and FVB to regions of the nucleus. LADs = laminar-associated domains (repressive state).

To determine whether this mechanism might be contributing to the difference in *Arntl2-*mediated metastasis efficiency, *in vitro* immunoprecipitation was repeated for validation. An increased binding of PARP1 to the MOLF promoter compared to the FVB sequence was observed (Fig 5A), as expected from the initial pull-down experiment. Additionally, an increased interaction was also found between the MOLF promoter and CTCF, consistent with the hypothesis that the MOLF *Arntl2* promoter might be more efficiently recruited to the transcriptionally repressive nuclear lamina than the FVB allele. To confirm that the differential binding of CTCF and PARP1 to MOLF was not due to uneven loading, we probed for RRP1B since it was shown to bind both promoters with equal affinity in the mass-spectrometry analysis. As shown in Fig 5A, there were no differences in the binding of MOLF and FVB to RRP1B, further demonstrating the stronger interaction of the MOLF promoter to the CTCF/PARP1 complex.

Recruitment of the *Arntl2* promoter to the transcriptionally repressive nuclear lamina might be expected to result in decreased expression of genes flanking the promoter by formation of a lamina associated domain (LAD). To determine if the expression of the genes flanking *Arntl2* are also altered due to chromosomal positioning, we performed qPCR of the flanking *Stk38l* and *Smco2* genes in the CRISPR-engineered cell lines. *Smco2* was not detected, suggesting that this gene is not expressed in our cell lines. However, *Stk38l* mRNA expression decreased in the heterozygous CRISPR 6DT1 cells compared to wildtype (Fig 5B) supporting the hypothesis that the chromosomal locus on which these genes lie may be undergoing a change in intra-nuclear positioning that results in transcriptional repression of the genes (Fig 5C).

## Discussion

Metastasis is a highly complex process that requires the completion of a series of sequential steps to enable a tumor cells to successfully colonize a distant organ. These steps include acquisition of invasive and migratory abilities, penetration into the lymphatics and/or vasculature, surviving the shear forces and anchorage independent state during transit and initial arrest at the secondary site, extravasation out of the circulatory system into the secondary site parenchyma, and finally colonization and growth in an ectopic environment [25]. In addition to the plasticity of tumor cell-intrinsic processes required to complete these steps, it has become increasingly clear over the recent years that tumor non-autonomous factors including the immune system and distant tissues such as the bone marrow also play critical roles in the metastatic cascade [26]. As a result, factors that influence many different mechanisms or biological processes can have either positive or negative impacts on the ability of a tumor to generate life-threatening metastatic lesions.

One of the factors that is frequently not considered in studies of metastasis is the influence of the genetic background [27]. Polymorphisms within populations are known to be associated with many disease conditions, including cancer, and can affect both cell autonomous and non-autonomous systems. Unlike cancer driver events, which are frequently somatically acquired missense or nonsense mutations that activate or inactivate oncogenes or tumor suppressors, polymorphisms associated with disease are usually non-coding and thought to be associated with variations in gene expression and pathway balance rather than constitutive gene activation or ablation. Disease-associated polymorphisms may therefore mark biological systems that might be exploited by more subtle manipulation than complete or near-complete suppression of activated oncogenes or their downstream effectors.

In this study we provide evidence that polymorphisms that affect the expression levels of *Arntl2*, a gene in the circadian rhythm pathway, has a significant effect on metastatic progression in ER- breast cancers. Previous studies have demonstrated an increased risk of breast cancer in women with circadian rhythm interruption/polymorphisms [28, 29]. Some evidence has implicated circadian rhythm gene expression with metastasis-free survival in breast cancer, but the majority of the associations were not independent of other clinical variables [30]. In addition, these results did not address a causal role for circadian rhythm genes or the mechanisms of gene expression variation across the patient samples. Here we directly tested the causal role of *Arntl2* using a variety of orthogonal methods. The results obtained are consistent with variation in *Arntl2* expression due to polymorphisms in the proximal promoter, although we cannot presently rule out contributions from additional variants in more distant regulatory elements. In addition to direct effects on transcriptional efficiency, differential binding of PARP1 and CTCF to the putative promoter variants suggests that the polymorphisms may play a role in modifying higher order chromatin biology. A recent study demonstrated that CTCF and PARP1-bound regions of chromatin oscillate between the transcriptionally active nuclear interior and the transcriptionally repressive nuclear lamina compartments during the circadian cycle [24]. Polymorphisms that affect the binding of these two chromatin modifiers might therefore affect the efficiency of recruitment to or release of the *Arntl2* promoter from the nuclear envelope, which in turn might alter the timing of *Arntl2* expression and its downstream target genes. This possibility is particularly intriguing due to the association of other metastasis susceptibility genes with the nuclear envelope (*Sipa1*, *Brd4* isoform 2; [31, 32]). Further studies will be required to assess this possibility as well as the role of *Arntl2* target genes in breast cancer metastatic progression.

In concordance with our findings, a recently published study by Brady et al. also identified *Arntl2* to be a metastasis promoter gene [33]. In this manuscript, the authors identify a secretory mechanism that drives metastatic self-sufficiency and enables lung adenocarcinoma cells to colonize and proliferate at the secondary site. Currently it is unclear whether or not this mechanism is also important in breast cancer. Previous analysis of transcription-based prognostic signatures in our lab has indicated that tissue-specific difference in metastasis biomarkers exist, suggesting different molecular mechanisms may be involved. Further investigations into the downstream mechanisms of Arntl2-mediated mammary metastatic progression are required to address this question and will be part of our future research endeavors.

Finally, this study highlights the utility of incorporating polymorphism analysis with genetically engineered mouse models in studies of metastatic disease. Genetically engineered mouse models have been, and will continue to be important to our understanding of the etiology of human disease. However, as demonstrated here, incorporation of complex genetic architecture into an individual model enhances the ability of the model to represent human disease. In this case, the introduction of the polymorphic content from the Asian mouse strain MOLF/EiJ enabled the MMTV-PyMT mouse model, thought to be a model of ER^+^-like luminal tumors [8, 9], to at least partially model the biology of ER^-^ breast cancers. While all models of human disease are at some level imperfect, introduction of polymorphism into the MMTV-PyMT and other models may result in a more accurate representation of the biology of disease that is seen in the genetically complex and diverse human population.

This study also highlights additional difficulties in interpretation of quantitative trait genetics in cancer biology. *Arntl2* was identified as a potential gene of interest based on 1) its presence at the apex of a QTL peak that spans the distal third of chromosome 6, and 2) gene expression associations with metastatic disease. The initial identification of *Arntl2* suggested that this gene would function as a metastasis-protective factor, based on both the mouse and human gene expression patterns. However, manipulation of *Arntl2* as a single factor in both cell lines and knockout animals consistently supported the role of *Arntl2* as a metastasis-promoting, rather than protective, factor. The most plausible explanation for this discrepancy is the presence of an additional gene or genes within the interval that interact with *Arntl2*, resulting in a prognostic phase inversion. Additional mammary cancer genes exist within the chromosome 6 interval since the locus was also associated with tumor latency and burden, neither of which was significantly affected by *Arntl2*. Multiple genes within a QTL interval affecting the same trait has also been observed [34], including metastasis modifying genes in the MMTV-PyMT model system ([10], [35]). These factors indicate that careful validation and interpretation is necessary for the dissection of metastasis-associated QTLs to appropriately interpret how genes individually and collectively contribute to the complex metastatic cascade.

Overall, this study has important implications regarding the role of the circadian rhythm in cancer progression and provides a potential mechanism to explain the previously suggested increased risk of breast cancers in nightshift workers. Furthermore, this provides the first evidence that transcriptional control elements can be engineered using CRISPR/Cas9 to establish the causative role of SNPs in inherited susceptibility to cancer metastasis. Further studies into the downstream targets of *Arntl2* will be needed to identify the exact mechanisms by which *Arntl2* modulates breast cancer cell metastasis.

## Materials and Methods

### MOLF/EiJ backcross analysis

The generation of the FVB/NJ x [MOLF/EiJ x MMTV-PyMT] backcross was performed as previously described (Cancer Res December 15, 2001 61; 8866). Genotyping was performed by the Center for Inherited Disease Research (http://www.cidr.jhmi.edu/). QTL mapping was performed with the R/QTL program using the J/QTL interface [15]. QTL peaks were considered significant if the p value was less than 0.05 after 10,000 permutations of the data to correct for genome-wide significance.

### Ethics statement

The research described in this study was performed under the Animal Study Protocol LCBG-004, approved by the NCI Bethesda Animal Use and Care Committee. Animal euthanasia was performed by cervical dislocation after anesthesia by Avertin. The Shanghai Breast Cancer Study and Shanghai Breast Cancer Survival Study were approved by the institutional review boards of Vanderbilt University, the Shanghai Cancer Institute, and the Shanghai Center for Disease Prevention and Control, and written informed consent was obtained from all participants.

### ARNTL2 knock-out mice

The *Arntl2* knockout mouse strain used for this research project was created from ES cell clone (Arntl2_F05) originally generated by the Wellcome Trust Sanger Institute. ES cells containing a targeted, non-conditional allele (tm1e(KOMP)Wtsi) for Arntl2 were obtained from the KOMP (Knock Out Mouse Project) repository and knock out mice were generated in a C57BL/6 background by SAIC-Frederick (Frederick, MD). Mice were genotyped using cassette-specific primers.

### Cell lines and cell culturing

Mouse mammary carcinoma cell lines 4T1 and 6DT1 were a generous gift from Dr. Lalage Wakefield (NCI, Bethesda, MD). All cell lines were cultured in Dulbecco’s Modified Eagle Medium (DMEM), supplemented with 10% Fetal Bovine Serum (FBS), 1% Penicillin and Streptomycin (P/S) and 1% Glutamate, and maintained in 37°C degrees with 5% CO2. Short hairpin RNA (shRNA)-mediated knockdown and overexpression cells were cultured in the same conditions with an addition of 10ug/ml puromycin and 5ug/ml blasticidin, respectively.

### Plasmid constructs

#### shRNA constructs

TRC lentiviral shRNA constructs against *Arntl2* were obtained from Open Biosystems (now Dharmacon) as glycerol stocks (RHS4533-EG56938). The sequences for all shRNA constructs were as follows:

C7- AAGTTTGTCCAGTTTACGCGC

C8- ATTCCAACAATATTTGGAGGC

C9- TTGTGAAACTAAACCATTGGC

C10- TTGAAAGAAGATAGGTAAGGG

C11- ATCTGTTCCAATACTACCAGC

*Overexpression*. MGC Mouse Arntl2 cDNA was purchased from Open Biosystems (now Dharmacon) as a glycerol stock (Accession: BC108965 Clone ID: 40047159). Using directional TOPO-cloning primers Fwd- 5’-CACC-ATGGAGTTTCCAAGGAAACGCA-3’ and Rev- 5’-GAGTGCCCACTGGATGTCAC-3’, Arntl2 cDNA was amplified using Phusion polymerase. DNA was purified using QiaQuick Gel extraction kit (Qiagen) and ligated into a Gateway entry clone using the LR reaction according to the manufacturer’s protocol (Invitrogen).

### Virus transduction

1.1 x 10^6^ 293T cells were plated in 6 cm dishes 24 hours prior to transfection in P/S-free 10% FBS DMEM media. Cells were transfected with 1ug of shRNA/cDNA and 1ug of viral packaging plasmids (250ng pMD2.G and 750ng psPAX2) using 6ul of Xtreme Gene 9 transfection reagent (Roche). After 24 hours of transfection, media was refreshed with 10% DMEM, supplemented with 1% P/S and 1% Glutamine. The following day, virus-containing supernatant was passed through a 45um filter to obtain viral particles, which were then transferred to 100,000 4T1/6DT1 cells. 24 hours post-transduction the viral media was removed and fresh 10% DMEM was added. Finally, 48 hours after transduction, the cells were selected with 10ug/ml puromycin- or 5ug/ml blasticidin-containing complete DMEM.

### Scratch assay

One day prior to scratch assay, 25,000 cells/well were plated in triplicates in an Essen ImageLock 24-well plate and allowed to grow to confluence. On the day of the assay, cells were treated with 10ug/ml Mitomycin C (Sigma) for 3–4 hours to inhibit cell proliferation. Scratch wounds were made using an Essen 4-channel scratch instrument loaded with Eppendorf 10uL micropipette tips and displaced cells and debris removed by washing with Phosphate-buffered saline (PBS) three times. The cells were placed into the IncuCyte Kinetic Live Cell Imaging System (Essen BioScience) in complete DMEM media and cell motility imaged for 24 hours.

### Cell proliferation assay

Cells were counted and 5000 cells/well were plated in quadruplicates in 24-well cell culture plates (Corning, Inc.) and placed into the IncuCyte Kinetic Live Cell Imaging System (Essen BioScience) and programmed to image each well at 2-hour intervals. Samples were imaged until they reached 100% confluence. Data analysis was conducted using IncuCyte 2011A software.

### RNA isolation, reverse transcription and Real-Time Polymerase Chain Reaction

RNA was isolated cell lines using TriPure (Roche) and reverse transcribed using iScript (Bio-Rad). Real-Time PCR was conducted using VeriQuest SYBR Green qPCR Master Mix (Affymetrix). Peptidylprolyl isomerase B (Ppib) was used for normalization of expression levels. Expression of mRNA was defined from the threshold cycle, and relative expression levels were calculated using 2- deltaCt after normalization with Ppib. Primer sequences were as follows:

*Arntl2* Fwd: GTCTTCCCCAGAATCCCTTT; *Arntl2* Rev: TTGTCTCTCCGACGCTTTTC *Stk38l* Fwd: TTCCTATGAGCAACCATACCCG; *Stk38l* Rev: TCTAGGCCAAGTCTGGTCCTC; *Ppib* Fwd: GGAGATGGCACAGGAGGAAAGAG; *Ppib* Rev: TGTGAGCCATTGGTGTCTTTGC

### Western blotting

Protein lysate from one million cells were extracted on ice using Golden Lysis Buffer (10 mM Tris pH 8.0, 400 mM NaCl, 1% Triton X-100, 10% Glycerol+Complete protease inhibitor cocktail (Roche), phosphatase inhibitor (Sigma)). Protein concentration was measured using Pierce’s BCA Protein Assay Kit and analyzed on the Versamax spectrophotometer at a wavelength of 560nm. Appropriate volumes containing 20ug of protein lysates combined with NuPage LDS Sample Buffer and NuPage Reducing Agent (Invitrogen) were run on 4–12% (or otherwise indicated) NuPage Bis-Tris gels in MOPS buffer. Proteins were transferred onto a PVDF membrane (Millipore), blocked in 5% milk (TBST + dry milk) for one hour and incubated in the primary antibody (in 5% milk) overnight at 4°C. Membranes were washed with 0.05% TBST (TBS + 5% Tween) and secondary antibody incubations were done at room temperature for one hour. Proteins were visualized using Amersham ECL Prime Western Blotting Detection System and Amersham Hyperfilm ECL (GE Healthcare).

The following primary antibodies were used: mouse anti-Actin (1:10,000; Abcam), mouse anti-myc-tag (1:1000; Cell Signaling). Secondary antibodies goat anti-rabbit (Santa Cruz) and goat-anti-mouse (GE Healthcare) were used at concentrations of 1:10,000.

### CRISPR cell lines

Single-guided RNA (sgRNA) against the *Arntl2* regulatory region was designed using the Massachusetts Institute of Technology CRISPR algorithm (crispr.mit.edu). The top sgRNA with the following binding sites was selected: 5’-GGAATCCCCCTCGCGACCGT-3’. sgRNAs were annealed and ligated into the pSpCas9(BB)-2A-Puro (PX459) (Addgene; 48139) vector (Nat Protoc. 2013 Nov;8(11):2281–308. doi: 10.1038/nprot.2013.143).

To create single CRISPR clones, 6DT1 cells were plated in 10cm dishes and transfected with 5ug sgRNA-containing PX459 vector and 5ug of linearized 900bp promoter region. Cells were transfected with Xtreme Gene 9 and 24 hours after transfection, cells were FACS-sorted by GPF fluorescence. A total of 1000 cells were sorted and single cell clones were manually plated into 96-well plates. Clones were allowed to grow up until they reached confluence and then gDNA was isolated to check for integration of the MOLF region by PCR-amplification with the following primers: Fwd- 5’-TAAGCA-ACGCGT-GGGCTGGCTAGGGCTG-3’ and Rev- 5’- TGCTTA-AGATCT-TACAAGAGAGTTGACAGGTCCAG-3’. PCR-amplified DNA was purified using QIAquick PCR Purification Kit (Qiagen) and digested using MseI at 37°C overnight.

### *In vivo* metastasis experiments

Female virgin FVB/nJ or BALB/cJ mice were obtained from Jackson Laboratory at 6–8 weeks of age. Two days prior to in vivo experiments, cells were plated at one million cells/condition into T-75 flasks (Corning) in non-selective DMEM. A total of 100,000 cells per mouse was injected into the fourth mammary fat pad of FVB/nJ or BALB/cJ mice for 6DT1 or 4T1 cells, respectively. For experimental metastasis assays, 100,000 4T1 tumor cells were injected into the tail vein of BALB/cJ mice. The mice were euthanized between 28–30 days post-injection. Primary tumor was resected, weighed and lung metastases counted.

### *In vitro* pull-down

The MOLF and FVB promoter regions were amplified using strain-specific gDNA and the following primers: Fwd /5BiosG/GAAGGTCCACACCCTCTTGC and Rev- /5BiosG/CCTGGACTTGGCCATTGGAA. Phusion taq polymerase was used according to the manufacturer’s recommendation with the following PCR conditions: 98°C for 15 sec, 35 cycles of 98°C for 10 sec, 72°C for 1.5 min followed by 72°C for 10 min and a final step of holding in 4°C. PCR products were purified and 5ug of DNA was used for each pull-down experiment along with nuclear lysate from 6DT1 cells (FVB background).

Nuclear lysate was isolated using Pierce’s NER Extraction kit according to the provided protocol. Following nuclear extraction, 200ug of lysate was incubated with 5ug of biotinylated DNA probes along with 100ul of streptavidin magnetic beads (Dynabeads M270, Thermo-Fisher (former Invitrogen)). The final volume of 500ul was adjusted using NER buffer from the extraction kit. The mixture was placed on a rotator and incubated at room temperature for 1 hour ([36]). The samples were placed on a magnetic stand and washed with ice cold PBS three times followed by one wash with NER buffer. The beads were resuspended in 25ul of 2x SDS buffer, boiled at 95°C for 5 min and the proteins were separated on a 4–12% SDS-Page minigel (Invitrogen). The gel was stained using Pierce’s Silver Stain for Mass Spectrometry according to the manufacturer’s protocol. Protein bands were sent to the ATRF facility at NCI–Frederick and identified via mass spectrometry.

For validation of the pulldown, the same conditions were used to bind the probes to the beads. The beads were washed with PBS three times followed by one wash with NER buffer. The proteins were separated on a 7% Tris-acetate SDS-Page minigel (Invitrogen) and transferred onto a PVDF membrane at 4°C for 1 hour. The membranes were probed with the following antibodies overnight at 4°C: rabbit-anti-PARP1 (Santa Cruz; 1:5000), rabbit-anti-RRP1B (Santa Cruz; K-19: 1:1000) and rabbit-anti-CTCF (Cell Signaling; 1:1000). Secondary antibody goat anti-rabbit (Santa Cruz) was used at concentrations of 1:10,000 at room temperature for 1 hour.

### Epidemiology studies

Samples included in the epidemiological study came from participants of the Shanghai Breast Cancer Study (SBCS) and Shanghai Breast Cancer Survival Study (SBCSS). Details on the methodology of the parent studies have been described previously [37–39]. Briefly, the SBCS is a population-based, case-control study that recruited 3,448 incident breast cancer patients and controls in urban Shanghai between August 1996 and March 1998 and again between April 2002 and February 2005; 90.6% of participants provided a blood or exfoliated buccal cell sample [37]. The SBCSS, also conducted in urban Shanghai, recruited 5,042 breast cancer patients between March 2002 and April 2006 with 98% of patients providing an exfoliated buccal cell sample [38]. All participants of both studies provided written informed consent before participating in the study and the Institutional Review Boards of all institutes involved approved the study protocols. Medical charts for breast cancer patients were reviewed to verify cancer diagnosis and obtain tumor characteristic (including estrogen receptor, ER) and treatment information. Cancer patients have been followed for survival status and breast cancer recurrence through a combination of record linkages with the Shanghai Vital Statistics Registry and in-person surveys. A total of 1,758 women with estrogen receptor negative breast cancer and DNA samples from the SBCS and SBCSS were included in the current study. Among them, 726 samples were genotyped using the Affymetrix Genome-Wide Human SNP Array 6.0 [39] and imputed according to the 1000 Genomes Project Asian data. Genotype from the remaining 1032 samples were genotyped using the iPLEX Sequenom MassARRAY platform. A total of 137 disease progression events were observed in the GWAS patients and 193 events were observed in the Sequenom genotyped patients.

## Supporting Information

S1 Fig *In vitro* effect of Arntl2. (A) Cell proliferation assay of Control and Arntl2 knockdown 4T1 cells as measured by confluency in the IncuCyte. (B) Wound closure of Control and Arntl2 knockdown 4T1 cells. (C) Relative mRNA expression of 4T1 cells transduced with control vector (8166) and Arntl2 overexpression (OE) vector as measured by qPCR. (D). Western blot of 4T1 cells transduced with control vector (8166) and Arntl2 overexpression (OE) myc-tagged vector. Actin serves as a loading control.
(PDF)

S2 Fig Analysis of SNPs within DHS site of Arntl2. (A) SNP differences between MOLF/EiJ and FVB/NJ in a DHS site 10kb upstream of Arntl2. Image depicts a screen shot of the UCSC Genome browser. (DHS = DNase hypersensitivity site) (B) Primer design for PCR of Arntl2 transcript (left). On the right is the gel image of the PCR product of 4T1 cells showing a 2kb band (arrow).
(PDF)

S3 Fig Silver stain of *in vitro* pulldown of MOLF and FVB promoter probe for mass spectrometry. Red circles indicate areas with most differences between MOLF and FVB.
(PDF)

S1 Table Gene list within QTL with significantly different expression. (PDF)

S1 File List of proteins identified from in vitro pulldown assay. (XLSX)
