# Supplementary figures and images for: The Circadian Rhythm Gene Arntl2 Is a Metastasis Susceptibility Gene for Estrogen Receptor-Negative Breast Cancer
Source: PLoS Genet. 2016 Sep 22;12(9):e1006267. doi: 10.1371/journal.pgen.1006267 (PMC5033489; doi:10.1371/journal.pgen.1006267)

Supplemental Figure 1

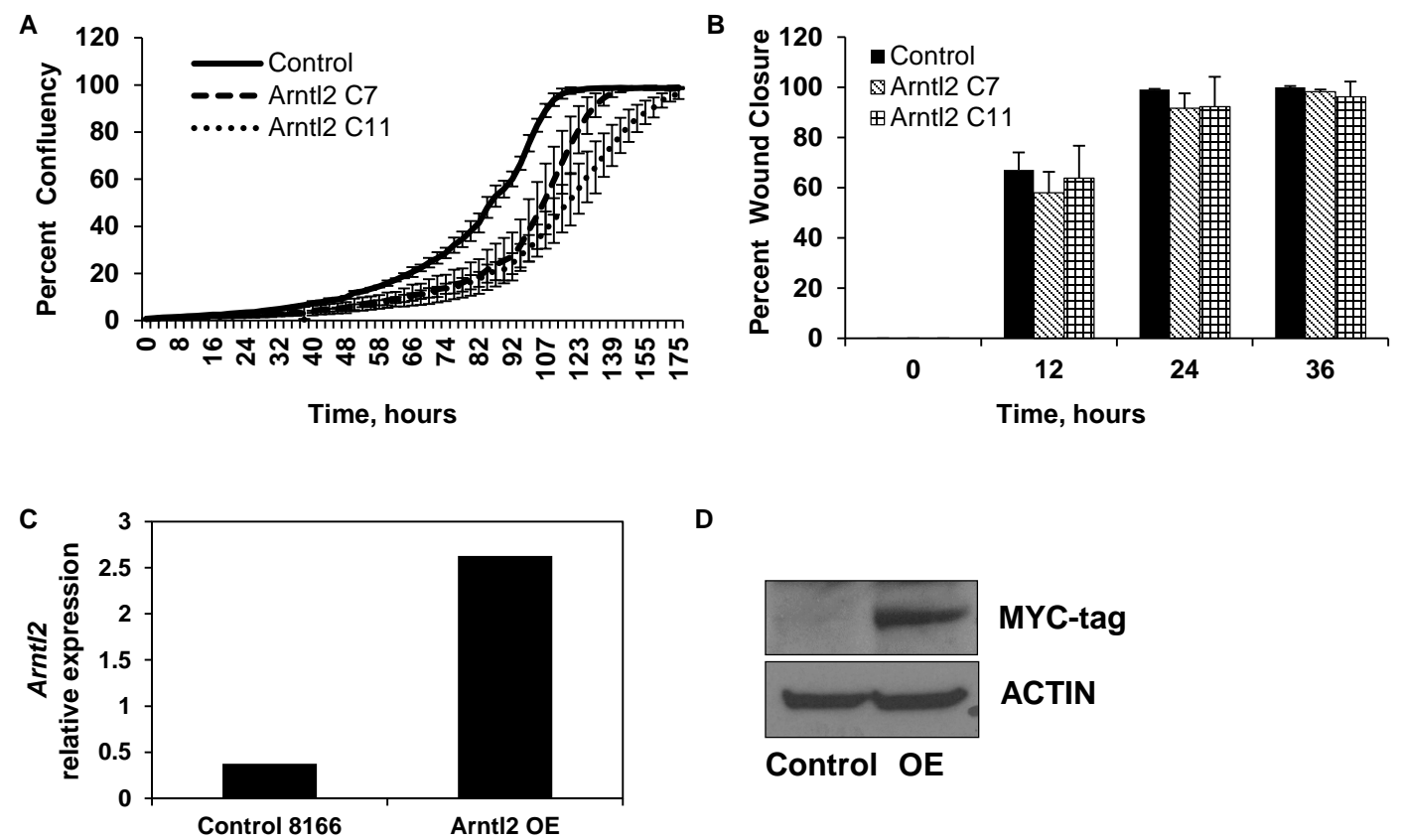

Supplement: S1 Fig — (A) Cell proliferation assay of Control and Arntl2 knockdown 4T1 cells as measured by confluency in the IncuCyte. (B) Wound closure of Control and Arntl2 knockdown 4T1 cells. (C) Relative mRNA expression of 4T1 cells transduced with control vector (8166) and Arntl2 overexpression (OE) vector as measured by qPCR. (D). Western blot of 4T1 cells transduced with control vector (8166) and Arntl2 overexpression (OE) myc-tagged vector. Actin serves as a loading control. (PDF) [file pgen.1006267.s001.pdf]

Supplemental Figure 2

A

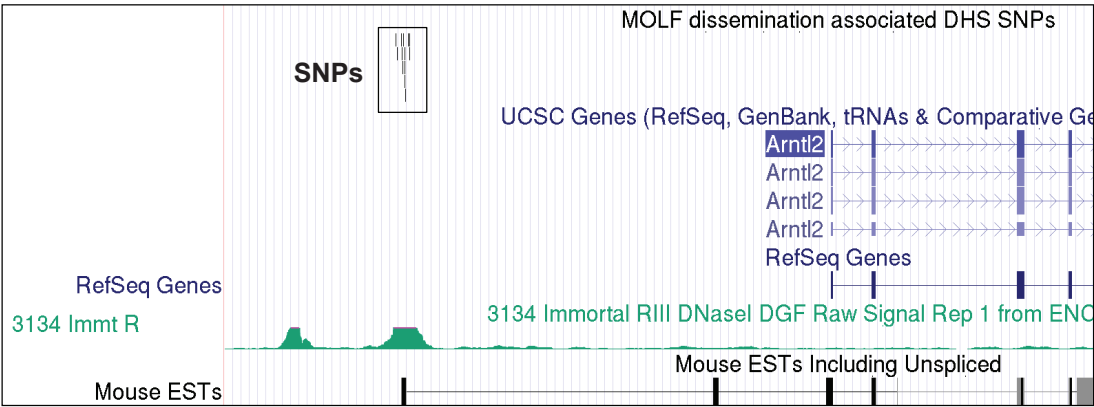

B

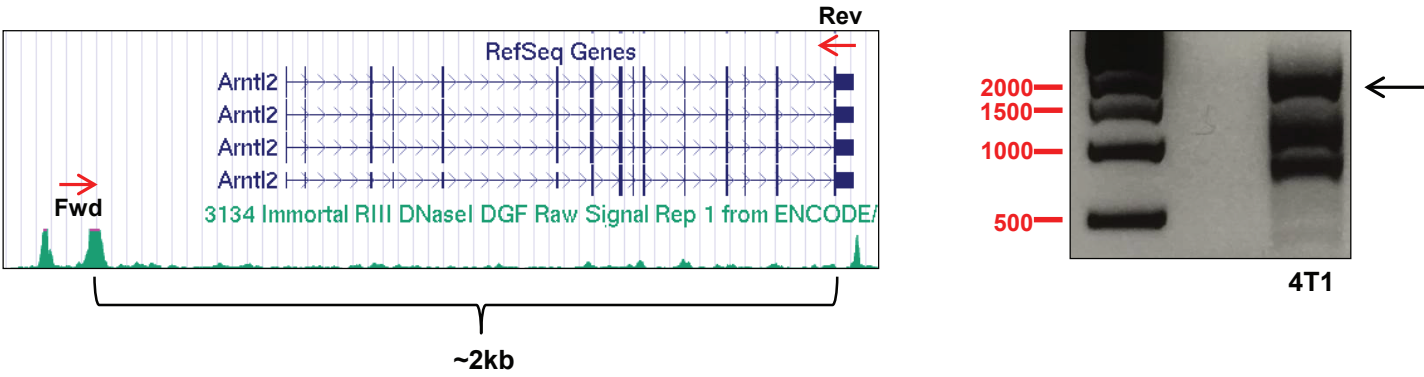

Supplement: S2 Fig — (A) SNP differences between MOLF/EiJ and FVB/NJ in a DHS site 10kb upstream of Arntl2. Image depicts a screen shot of the UCSC Genome browser. (DHS = DNase hypersensitivity site) (B) Primer design for PCR of Arntl2 transcript (left). On the right is the gel image of the PCR product of 4T1 cells showing a 2kb band (arrow). (PDF) [file pgen.1006267.s002.pdf]

Supplemental Figure 3

A

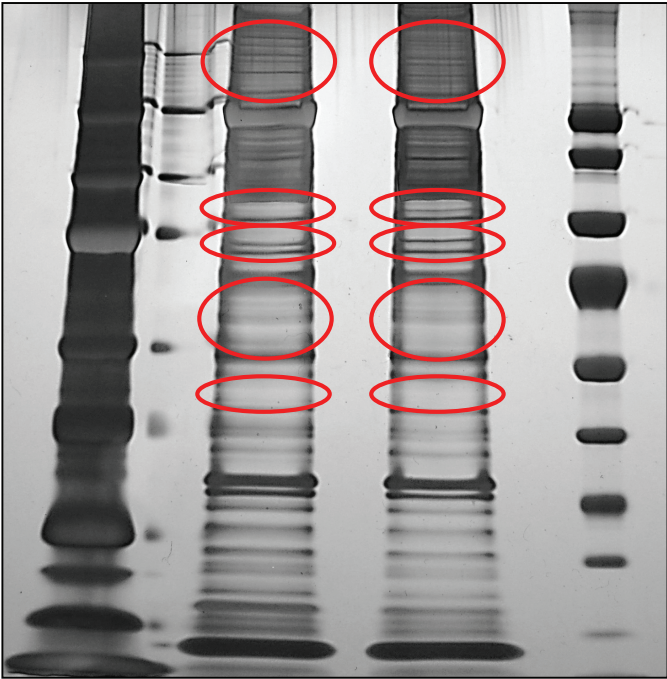

MOLF  
promoter  
probe

FVB  
promoter  
probe

Supplement: S3 Fig — Red circles indicate areas with most differences between MOLF and FVB. (PDF) [file pgen.1006267.s003.pdf]
