# Supplementary material for: The Circadian Rhythm Gene Arntl2 Is a Metastasis Susceptibility Gene for Estrogen Receptor-Negative Breast Cancer
Source: PLoS Genet. 2016 Sep 22;12(9):e1006267. doi: 10.1371/journal.pgen.1006267 (PMC5033489; doi:10.1371/journal.pgen.1006267)
Supplement: S1 Table — (PDF) [file pgen.1006267.s004.pdf]

Supplemental Table 1

Gene Symbol	Hazard Ratio	P value
**Arntl2**	0.26	< 1e-07
**2810474O19**	0.2	< 1e-07
**4833442J19**	0.17	< 1e-07
**Clec2g**	0.03	< 1e-07
**Il17re**	14.06	< 1e-07
**Fgfr1op2**	0.13	< 1e-07
**Osbpl3**	6.2	< 1e-07
**Clec7a**	0.12	< 1e-07
**Slco1a5**	1.8	< 1e-07
**Pik3c2g**	0.38	< 1e-07
**Ephb6**	4.2	7.00E-04
**Emp1**	3.4	0.0011
